# Sustainable Silk-Based Particulate Systems for the Controlled Release of Pharmaceuticals and Bioactive Agents in Wound Healing and Skin Regeneration

**DOI:** 10.3390/ijms25063133

**Published:** 2024-03-08

**Authors:** Beatriz G. Bernardes, Anabela Veiga, Joana Barros, Carlos A. García-González, Ana Leite Oliveira

**Affiliations:** 1 Universidade Católica Portuguesa, CBQF—Centro de Biotecnologia e Química Fina—Laboratório Associado, Escola Superior de Biotecnologia, Rua Diogo Botelho 1327, 4169-005 Porto, Portugal; bbernardes@ucp.pt (B.G.B.); s-anveiga@ucp.pt (A.V.); 2 AerogelsLab, I+D Farma Group (GI-1645), Department of Pharmacology, Pharmacy and Pharmaceutical Technology, iMATUS and Health Research Institute of Santiago de Compostela (IDIS), Universidade de Santiago de Compostela, E-15782 Santiago de Compostela, Spain; 3 LEPABE-Laboratory for Process Engineering, Environment, Biotechnology & Energy, Department of Chemical Engineering, Faculty of Engineering, University of Porto, R. Dr. Roberto Frias, 4200-465 Porto, Portugal; 4 ALiCE-Associate Laboratory in Chemical Engineering, Faculty of Engineering, University of Porto, Rua Dr. Roberto Frias, 4200-465 Porto, Portugal; 5 i3S-Instituto de Investigação e Inovação em Saúde, Universidade do Porto, R. Alfredo Allen 208, 4200-135 Porto, Portugal; joana.barros@ineb.up.pt; 6 INEB-Instituto de Engenharia Biomédica, Universidade do Porto, R. Alfredo Allen 208, 4200-135 Porto, Portugal

**Keywords:** silk proteins, drug carriers, particulate systems, wound healing, tissue engineering

## Abstract

The increasing demand for innovative approaches in wound healing and skin regeneration has prompted extensive research into advanced biomaterials. This review focuses on showcasing the unique properties of sustainable silk-based particulate systems in promoting the controlled release of pharmaceuticals and bioactive agents in the context of wound healing and skin regeneration. Silk fibroin and sericin are derived from well-established silkworm production and constitute a unique biocompatible and biodegradable protein platform for the development of drug delivery systems. The controlled release of therapeutic compounds from silk-based particulate systems not only ensures optimal bioavailability but also addresses the challenges associated with conventional delivery methods. The multifaceted benefits of silk proteins, including their inherent biocompatibility, versatility, and sustainability, are explored in this review. Furthermore, the intricate mechanisms by which controlled drug release takes place from silk-based carriers are discussed.

## 1. Introduction

Drug carriers are designed for the transportation of bioactive agents and other molecules in pharmaceutical, cosmetic, and nutraceutical applications [1,2]. The effectiveness of a drug carrier relies on the presence of a robust protective material for the bioactive agent, protecting it from physiological environments and resisting mass flow and diffusion from the material to the external bulk of the system [1]. Drug carriers can be administered through oral, nasal, pulmonary, ocular, transdermal, subcutaneous, anal, transvaginal, and intravenous administration routes [1,3]. The selection of the administration route is critical, as it influences the activity of the active compound, subjecting it to different physiological environments within the human body. Depending on the viscosity of the medium, the drug release from the carrier material also exhibits differing kinetic profiles. Therefore, an additional factor influencing the characteristics of the drug carrier is the nature of the surrounding medium, which may consist of water, gel, or a blood-like medium [1]. The chemical structure of the carrier can be modified to adapt to the administration route, but the influence of this change on the stability and effectiveness of the bioactive agent to interact with target tissues should be assessed as it may affect its intended function [4].

Some of the earliest recorded medicinal treatments were administrated topically. Skin, our body’s largest organ, while playing a crucial role in protecting internal organs by serving as a barrier against the external environment and harmful pathogens, is permeable to certain therapeutic molecules. These compounds, applied or bandaged onto the skin, addressed diverse skin issues with tailored substances and natural materials, such as oils, clays, yogurt, and herbal extracts, serving specific purposes like protection of wounds, cleansing, exfoliation, and moisturization [5]. Among these, silk has been used for centuries in wound healing applications (suturing) due to its biocompatibility, lack of immunogenicity, and ability to be naturally absorbed [6]. In modern medicine, silk has gained increased attention over the years. The inherent ability of silk to stimulate cell migration and proliferation is directly associated with its accelerated wound-healing properties. Silk proteins are suitable as a biomaterial for developing wound dressings and bioartificial skin grafts [6,7]. Another interesting example is the commercial product Silk Voice, a scaffold composed of reconstituted or solubilized silk protein, which received Food and Drug Administration (FDA) approval in 2019, with Sofregen Medical, Inc. overseeing the product for augmenting the size of the displaced or deformed vocal fold.

Drug carriers have undergone extensive research as potential systems for wound healing treatment in more modern medicine. Various types of drug carriers, including hydrogels, scaffolds, particles, liposomes or transferosomes, complexes, and coacervates, have been studied for the controlled release of antibiotics, cellular growth factors, gene transfer, anti-inflammatory agents, and so on [8,9]. In recent years, innovative drug delivery systems for skin regeneration and wound healing, such as stem cell-based therapy, have been intensively studied [10]. Microcapsules, nanoparticles, microspheres, nanotubes, dendrimers, liposomes, polymeric micelles, and extracellular vesicles are among the carriers that can protect bioactive compounds, thereby avoiding or mitigating inadvertent administration to healthy tissues [1,4].

Particulate systems are important in wound healing because they can encapsulate bioactive compounds and release them at the wound site, opening different possibilities such as inflammation/infection control, stimulation of cell proliferation, and promotion of tissue regeneration. Their local delivery minimizes side effects, optimizes therapeutic efficacy, and allows easy handling, even in deep wounds [2]. This review centers on the exploration of silk-based particulate systems for local drug delivery in wound healing and skin regeneration. To our knowledge, this is the first review focusing on the study of micro- and nanoparticles, liposomes, and aerogel particles as silk-based drug carriers for wound healing and skin applications. These systems can provide a controlled release of pharmaceuticals and bioactive agents, making wound healing and regeneration technologies sustainable. The use of silk proteins, Silk fibroin (SF), and sericin (SS), known for their biocompatibility and eco-friendly properties, highlights the recent advancements towards finding sustainable approaches for healthcare problems.

### 1.1. Wound Healing and Skin Regeneration

Skin is the most complex and largest human organ, consisting of the epidermis, dermis, and skin appendages such as hair follicles and sebaceous glands. The complex process of wound healing entails multiple coordinated phases, encompassing hemostasis, inflammation, proliferation, maturation, and remodeling [2]. These phases are regulated by a range of cell types, such as macrophages, mast cells, fibroblasts, and keratinocytes, that result from immune system activation, the coagulation cascade, and inflammatory pathways. This is pivotal for achieving comprehensive restoration of all skin compartments, encompassing cellular proliferation and extracellular matrix (ECM) remodeling [2,11,12]. During wound healing, the body’s natural production of exudate, a fluid mixture of water, nutrients, electrolytes, proteins, and various cellular elements, creates an environment conducive to healing [2]. In chronic wounds, the delayed or prolonged healing process can result in scar formation, which can have a substantial impact on the aesthetics of the skin aspect and, therefore, the psychological well-being of patients, lowering their quality of life. The formation of scars hinders the complete restoration of skin function, underscoring the necessity of creating novel materials to expedite healing and prevent scar formation [2,12]. This highlights the importance of researching and developing biocompatible drug carriers as a primary method to improve the healing process and skin regeneration.

### 1.2. Silk Proteins as a Drug Carrier on Wound Healing and Skin Regeneration

**Figure 1:**
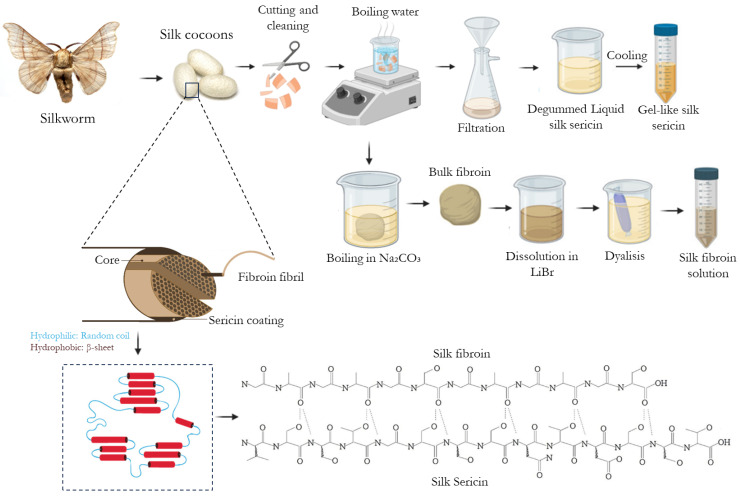
Schematic representation of silk organization, composition, and chemical structure (evidencing the intermolecular hydrogen both between fibroin and sericin) and the most used extraction methodologies of both silk proteins for biomedical applications (adapted from [18,19] (Creative Commons CC BY 4.0 license) and [20] (Copyright 2022 American Chemical Society)).

Natural-derived biopolymeric drug carriers have been studied for wound and skin regeneration because of their tunable physiochemical properties, such as biodegradability, biocompatibility, and hydrophilicity [2,13]. Among them, silk biomaterials have been studied for use in many biomedical applications, including drug delivery systems. Silk is a protein biopolymer with a unique set of properties that can be produced by a variety of insects, such as spiders, silkworms, bees, and others [7,14]. Silkworm silk, specifically derived from the domestic *Bombyx mori*, stands out as the most common silk source in the commercial silk industry due to its scalability [14]. Silkworm silk is composed of two proteins, SS and SF, accounting for 20–30% and 70–80% of the total, respectively, where SF is connected to SS, a gummed amorphous protein, within the silk fiber proteins [15]. These proteins have notable characteristics, including biocompatibility, regenerative potential, anti-inflammatory and antioxidant properties, and ease of functionalization, which involves modifying the surface chemistry of a material to introduce new functions, features, capabilities, or properties [16,17]. SS and SF are made up of repeated hydrophobic and hydrophilic domains (Figure 1). The hydrophobic sections create antiparallel β-sheet crystals to enhance strength, while the hydrophilic domains contribute to the formation of amorphous regions, promoting extensibility [14]. The alignment of the repeated silk sequence differs depending on the extraction source [14]. These characteristics make them excellent contenders for crafting biomaterials that not only elicit a favorable clinical response but also prove to be economically efficient [16].

#### 1.2.1. Silk Fibroin

SF is a one-of-a-kind natural protein as a potential biopolymer for biomedical applications [7,17,21]. Table 1 displays the outstanding features of SF for biomedical engineering.

SF primarily consists of hydrophobic β-sheet crystallites and hydrophilic amorphous domains [17,39]. The crystalline segments are made up of heavy- and light-chain polypeptides, with a molecular weight of 391.6 and 27.7 kDa, respectively [39]. These chains are linked by a single disulfide bridge as well as a glycoprotein named P25 (molecular weight of 25.4 kDa), which contributes to SF’s exceptional combination of properties such as strength, modulus, toughness, lightweight, elasticity, oxygen, and water vapor permeability, customizable degradability, and signaling molecule stabilization [17]. SF is largely composed of 43% glycine (Gly), 30% alanine (Ala), 12% serine (Ser), and threonine or valine [17,36]. The amino acid sequence of the heavy chain can be described as (-Gly-Ser-Gly-Ala-Gly-Ala-)_n_ [17,39]. The hydrophobic domains of the heavy chain contain a repetitive hexapeptide sequence capable of forming stable anti-parallel β-sheet crystalline structures. In contrast, the amino acid sequence of the light chain lacks repetition, making it more hydrophilic and relatively elastic within the structure of SF [17,36]. SF has been found to exhibit excellent mechanical properties [21].

To extract SF, the SS located in the outer layer of the fiber must be removed. The water-soluble nature of SS allows the use of different degumming methods without affecting the structure of SF. Silk degumming processes employ a variety of degumming methods, including natural degumming agents (e.g., mixed incorporation of several proteases), chemical (e.g., organic acids, sodium carbonate, oxidation process, supercritical fluid with carbon dioxide, etc.), or physical processes (steam treatment, high temperature, microwave irradiation, etc.) [15]. One of the most predominant degumming processes employed in the extraction of SF entails the removal of SS by boiling the silkworm cocoon in a solution of sodium carbonate [17,40,41,42]. Subsequently, the SF fibers undergo washing and drying. The dissolution of these dried fibers occurs in a 9.3 M lithium bromide solution, followed by a dialysis process [17,19,36]. The molecular weight of SF is strongly affected by the time of the degumming process and the concentration of sodium carbonate solution [36,43]. A shorter degumming period tends to result in SF with a high molecular weight, while a longer degumming time tends to reduce molecular weight, allowing for easy manipulation of drug release behavior from SF-based matrices [36,44]. Along with facile molecular weight manipulation, SF crystallinity can also be easily tuned. Induced crystallinity is a crucial stage in the formation of SF-based scaffolds, microparticles, hydrogels, and films and can be altered to control drug release rates [45]. Traditional procedures, such as water annealing or immersion in ethanol or methanol, can be used to induce this [36].

#### 1.2.2. Silk Sericin

SS is often classified as either SS A, SS B, or SS C according to its location in the silk cocoon and its solubility. SS A forms the outer layer and dissolves in hot water; SS B is in the middle layer; and SS C is next to SF, which includes the inner layers [46]. This global protein comprises 18 amino acids, with 45.8% hydroxy amino acids (serine and threonine), 42.3% polar amino acids, and 12.2% non-polar amino acids [18,47,48,49]. SS structure includes random coil and β-sheet conformation, being mostly amorphous after dissolution (liquid) and more crystalline after cooling (gel-intrinsic properties). Changes from β-sheet to random coil occur as a response to mechanical stretching properties, moisture absorption, and temperature [22,50,51,52].

SS degumming can be achieved by using an infrared (IR) dyeing process, in which water molecules act as abrasives during energy transmission via electromagnetic waves [18,47,49]. Additionally, chemical reagents such as alkaline reagents (sodium carbonate and sodium hydroxide), acid reagents (citric acid and tartaric acid), and proteases (papain, bromelain, pancreatin, and fungal protease) are often employed to augment degumming efficiency [18,47,49]. Boiling silkworm cocoons in a sodium carbonate solution is a widely adopted laboratory degumming method, demonstrating high SS extraction efficiency [18,47,49]. However, this process is energy-consuming, and partial hydrolysis can occur as a result of the harsh processing conditions [50,51]. The high-temperature and high-pressure method (HTHP) can be used since it dissolves SS in heated water at high pressure, removing the need for complex refining and purification processes. However, this method often has low yields. SS extraction in boiling water is also a common approach, being considered a simpler, less expensive, and more environmentally friendly process [52,53]. It requires only water and minimal lab material, and residual amounts of waste are produced in the form of silk threads. SS obtained from this method is also considered nonmutagenic and nongenotoxic both in vitro and in vivo [54]. Moreover, concentration can be achieved by further evaporating the resulting SS solution [55]. Different molecular weights can be obtained depending on temperature and extraction time [56] but usually range from 25 to 440 kDa [18,55]. These can then be used to prepare drug delivery systems. SS can be administered orally without causing toxicity, as demonstrated in vivo (mice) at dosages up to 2000 mg/kg [57]. In this study, it was observed that following a 3 h simulated in vitro digestion, the molecular weight of SS decreased from 25 to 260 kDa to a range of 10–15 kDa. These findings suggest that SS with higher molecular weights can undergo digestion into smaller forms, indicating that the absorption and consumption of SS are unlikely to induce inflammation in humans [58].

Besides the interesting SS biological properties (Table 2), SS structure can be easily cross-linked and copolymerized, providing limitless opportunities for its integration with various molecular materials to create innovative, high-quality compounds (including hydrogels, scaffolds, and particles) for biomedical engineering applications [16].

### 1.3. Overcoming Challenges in Scaling up Silk-Based Materials for Industrial Applications

It is important to evaluate the suitability of silk materials for industrial-scale applications due to the limited natural sources of silk proteins, which present challenges. It is imperative to achieve large-scale production through cost-effective means for their use in the clinic. Two methods for silk protein synthesis stand out for their scalability: (1) silk fibroin extraction and (2) bioengineered silk fermentation (constructing, expressing, and purifying plasmids) [83]. These methods have emerged as the primary methods for silk protein synthesis at an industrial level.

Prathumpai et al. [84] developed a pilot-scale production of serine protease from *Bacillus* sp. C-4, which has been reported as a candidate protease in the degumming process of the silk industry. On a pilot scale, the authors developed a 300-litre reactor with batch feeding and achieved a substantial protease activity of 1260 U/mL (28 U/mL/h). The efficiency of the degumming process was approximately 81% when considering the alkaline degumming process. The protease can be conveniently preserved at room temperature for up to one month and remains stable when refrigerated for over two months without the need for chemical additives or cryoprotectants [84].

Regarding SS, most of the scientific findings were reported in the last decade, and its application for biomedical engineering is still in the initial stages of development when compared to other well-established protein-based materials [16]. The early attempts to employ SS in biomedical engineering failed due to immunological response and cytotoxicity [85]. This phenomenon was later attributed to a lack of suitable extraction and purification techniques [86]. Until recently, SS was regarded as a byproduct of the textile industry, with tons of leftovers produced each year since the chemical oxygen demand and SS content of the silk degumming wastewaters may be as high as 60 g/L and 30 g/L, respectively [87]. This contributes to water pollution, potentially impacting aquatic ecosystems and a significant economic value [88]. The cost of commercial SS fluctuates based on its intended application, spanning from 40 €/kg for ISO9001 Certified SS to 100 €/g for dry SS, such as Sigma-Aldrich (St. Louis, MO, USA) SS *Bombyx mori*. The pricing is influenced by factors such as physical properties and purity [87].

Nowadays, different protocols have been applied to degum SS to obtain a biocompatible protein source for the development of new biomaterials.

However, the literature still lacks information about the effect of different concentration methodologies on the final properties of SS, standardization protocols, and preservation of its gelling properties, which are useful for the development of gels and three-dimensional hydrogels for TE. Recently, our research group proposed a new cryo-based methodology to obtain a sterile and off-the-shelf SS powder that can be dissolved in boiling water to obtain a gel-like structure. These findings open several possibilities for the development of new SS-based materials [89]. In addition, the off-the-shelf nature of the SS powder addresses one of the key logistical challenges in the industrial application of biomaterials: storage and transportation. This ready-to-use format significantly streamlines the manufacturing process, reducing the complexity and time required to prepare SS-based materials.

## 2. Particulate Drug Delivery Systems in Wound and Skin Regeneration

### 2.1. Silk-Based Microparticles

#### 2.1.1. Silk Sericin Micro-Systems

Silk-based microparticle systems have been explored over the last decades as a result of their stability, drug-loading capacities, ease of handling (often compared with conventional manufacturing processes), tissue penetration, and ease of functionalization [90].

**Figure 2:**
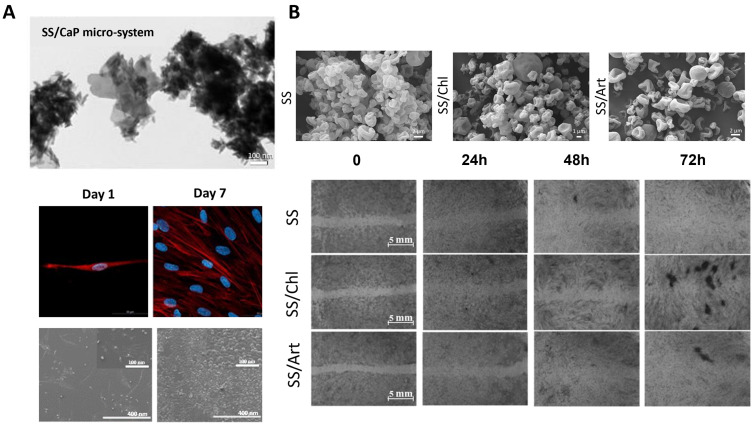
SS-based microparticle systems: (**A**) SS/Calcium Phosphate (CaP) particles and human dermal fibroblast cell response after direct contact (adapted from [92]), (**B**) SS/microalgae (Chl, Art) particles and wound healing capacity over time of human dermal fibroblasts (adapted from [94], Copyrights 2017 by [94]. Licensee MDPI, Basel, Switzerland).

As an organic natural material, SS can provide different functionalities, including antioxidant and other bioactive properties, particle modulation, and assembly mechanisms. To study the biomineralization mechanism of calcium phosphate (CaPs), SS can be used as the organic component in the organic/inorganic bone-like structures by adding it to a precipitation system. The production of these particles has been carried out in a stirred tank batch reactor [53] and in a meso-oscillatory flow reactor in batch [52]. Even though the individual particles formed were on the nanoscale (0.77 µm), the aggregation effect of CaPs resulted in a micro-cluster of hybrid particles. Additionally, it was shown that the introduction of SS helps modulate CaPs mineralization, affecting its physicochemical properties. The authors showed that increasing the SS concentration drives a rise in both the average particle size and the number of plate-like particles. Concurrently, the degree of aggregation increases while crystallinity decreases, resulting in composites with a bone-like inorganic/organic composition (80/20 wt.% CaPs/SS). More recently, the same constructs were fabricated using an advanced continuous fabrication process in a modular plate oscillatory flow reactor to guarantee scalability, enhanced efficiency, and reproductivity of these particles (Figure 2A) [72,91,92]. The particles were also validated for skin-tissue engineering (TE) by doing in vitro assays as a result of calcium’s role in promoting skin homeostasis and contributing to wound healing [93].

According to Bari et al., the aggregation of tiny nanoparticles can result in functional microparticle systems that provide biological cues [95]. In this study, a spray-drying approach was employed to generate smooth microparticles (1.7–2.30 µm) from an SS-based dispersion crosslinked with crocetin. All the formulations could prevent oxidative stress damage on nucleus pulposus cells, especially microparticles with higher crocin/crocetin content. Moreover, the formulations containing excess SS or crocin/crocetin were shown to be the most active in antioxidant and anti-tyrosinase activities. Besides, the particles with an SS excess also had higher anti-elastase activity. Regarding the drug release profile, the dose-dependent antioxidant activity observed in SS/crocetin particles and SS/crocetin with excess SS formulations highlights the controlled release mechanism that potentially allows for sustained delivery of active ingredients. Moreover, SS/crocetin with excess crocetin showed maximum activity at the lowest concentration tested, suggesting an efficient release and potent activity of crocetin. Thus, this system was proposed as a suitable strategy for intervertebral disk degeneration [95].

Bari et al. [96] then studied the application of SS microparticle systems for low-impact and non-invasive treatment of intervertebral disk degeneration. This strategy consisted of mixing SS with growth factors (platelet lysate (PL) and platelet-poor plasma (PPP)) and producing smooth microparticles (2.28 ± 0.07 µm) using spray-drying. It was shown that the mixture of PL + PPP increases the proliferation of nucleus pulposus cells, leading to a noteworthy decrease in cellular doubling time. When used alone or in conjunction with PPP, SS microparticles exhibited elevated ROS-scavenging activity. Notably, the combination of SS microparticles and PL demonstrated superior effectiveness in shielding nucleus pulposus cells from oxidative stress induced by hydrogen peroxide. As a result, Bari et al. stated that SS microparticles and PL + PPP could be a useful approach for developing low-impact and non-invasive drug delivery systems for tissue regeneration and wound healing applications [96].

SS and PL have been employed for various reparative treatments and have been shown to promote the wound healing process by regulating fibroblast recruitment, migration, proliferation, and differentiation [49]. For these reasons, it was hypothesized that the combination of PL and SS could also be suitable to produce active wound-healing advanced particles [97]. For instance, different microalgae (*Chlorella vulgaris* and *Arthrospira platensis*, Chl and Art, respectively) were mixed with SS particles for the topical treatment of wounds [94]. The resulting spray-dried microparticles (2–3 µm) exhibited a spherical and rough surface that showed good cytocompatibility, promoting human fibroblast cell proliferation and migration and, as a result, a complete wound closure (Figure 2B). However, Art and SS/Art showed higher antioxidant properties than other formulations [94].

A water-in-oil emulsification method can be applied to develop SS-loaded microparticles. Phromsopha and Baimark [98] produced spherical-shaped chitosan/SS microparticles with a smooth surface (41–48 µm) and high loading efficiency (69–75%). SS is released from the system through an in vitro process in a phosphate buffer at pH 7.4 and 37 °C over 24 h. As the SS concentration increases, the antioxidant properties and release content also increase. After 6 h, the release percentages were 38%, 43%, and 48% for SS ratios of 1%, 5%, and 10% by weight. After the initial burst, a sustained release was verified. After 24 h, these release levels increased to 45%, 55%, and 72% [98]. This enhancement in antioxidant properties is beneficial for wound healing because antioxidants can reduce oxidative stress in the wound area, thereby improving the healing process by protecting cells from damage.

Delivery of antiviral drugs by using soluble SS microparticles was proposed in the investigation carried out by Chen et al. [99]. Antiviral drugs can play a crucial role in the wound-healing process, especially in wounds that are at risk of viral infections or are already infected [9]. SS-dextran conjugate microparticles were prepared by the reprecipitation method, in which the compound was added to deionized water and the solution was injected into vigorously stirred organic solvents using a microsyringe. The size and morphology of the microparticles could be adjusted by the solvents and at low concentrations. Heterogeneous microspheres could be prepared in ethanol with a range of 85–390 nm and hollow mesoporous microspheres in propanol with an average particle size of 2.5–22 µm. The in vitro release studies of atazanavir from the developed SS-dextran conjugate microparticles demonstrated varied release rates across different pH conditions. At pH 8.0, the release of atazanavir was the slowest, with 51.2% of the drug released after 120 h. In contrast, the release rates increased in more acidic environments, with 99.2%, 97.8%, and 94.8% of atazanavir released at pH 7.4, 6.5, and 2.0, respectively, over the same period. This pH-dependent release behavior indicates the microparticles’ potential for targeted drug delivery, leveraging the pH variations within different body compartments to optimize therapeutic effectiveness [99].

#### 2.1.2. SF Micro-Systems

As SF materials demonstrate enhanced mechanical properties, most SF micro-systems reported have structural functionality. According to recent evidence, most SF microparticles are produced by using a “top-down” or “bottom-up” methodology. In the papers of Nosenko [100] and Moisenovich [101], the same “top-down” approach was followed to obtain SF/gelatin microparticles for soft-TE. The microparticles were generated by cryo-destruction of SF composite sponge scaffolds substituted with 30% gelatin. SF-microparticles with a porous surface with a diameter of 100–300 µm were used for wound healing regeneration, and microparticles with 250–500 μm were applied in brain regeneration after injury.

**Figure 3:**
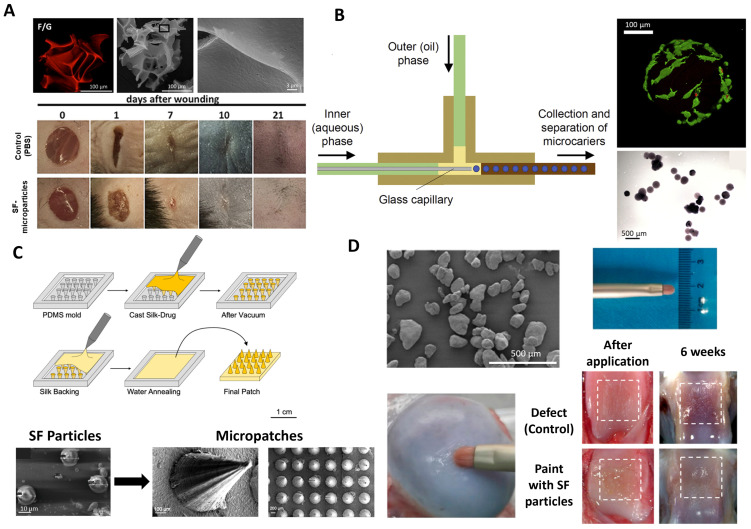
SF-based microparticle systems: (**A**) SF-based particles and applications for wound healing (adapted from [100], Copyright 2018 [100]), (**B**) SF-microparticles production using an axisymmetric flow-focusing device and biological response after culturing with rat mesenchymal stem cells (adapted from [41], Copyright 2019 [41]. Published by Elsevier B.V.), (**C**) SF microparticles incorporation in microneedle patches (adapted from [105], Copyright 2020 American Chemical Society) and (**D**) SF microparticles containing pain applied in cartilage defect over time (adapted from [106]. Copyright 2020 American Chemical Society).

In vitro studies for wound healing engineering showed that SF/gelatin microparticles, when placed in contact with a full-thickness mouse model, induced transient inflammatory gene expression and cytokine production. The ability of microparticle carriers of equal pro-regenerative potential, when compared to spidroin-based microparticles (main proteins in spider silk), to induce an inflammatory response may allow their subsequent adaptation to the treatment of wounds with different bioburden and fibrotic content [100] (Figure 3A). Primary cell cultures of neurons and astrocytes grown on SF/gelatin microparticles revealed greater viability under oxygen–glucose deprivation than two-dimensional conditions on plastic plates in the field of traumatic brain injury. Moreover, in vitro results obtained the first day after injecting the produced particles into a brain-damaged area showed that the material induces neuroprotection and recovery of long-term neurological functions [101].

For more long-term applications such as facial fillers, a “top-down” bioprinting process was implemented by Xie and the research team [102] using a glycidyl methacrylate-modified SF hydrogel. The hydrogel was printed, and the β-sheet content and mechanical properties were modulated by the number of freeze-thawing cycles. To obtain the particles, the material was pressed against stainless steel screens with different pore sizes (291–387 µm). The microparticles were dispersed in a hyaluronic acid solution and subcutaneously injected into the backs of the rats. After the injection, the size distribution of microparticles had no noticeable variation, suggesting that the microparticles could bear the shear strain without breaking during the injection. Moreover, the in vitro experiments demonstrated that the particles not only had desirable biocompatibility but also facilitated fibroblast migration, as well as blood vessel formation around the microparticles [102].

In “bottom-up” strategies, other strategies have been reported that include the use of different devices and equipment. In Luetchford et al. [41], SF/gelatin microparticles (300–400 μm with pores in the region of ~2 μm) were produced using an axisymmetric flow-focusing device (Figure 3B). The microparticles supported the adhesion of rat mesenchymal stem cells with high degrees of efficiency under dynamic culture conditions. After culturing in an osteogenic differentiation medium, cells showed osteoblast-like characteristics and osteogenic differentiation comparable to those of commercially available particles. The incorporation of gelatin in the microparticle system led to significantly improved cell response and increased Young’s moduli [41].

Spray-drying techniques can also be implemented to obtain SF microparticles. SF/chitosan particles loaded with recombinant human PTH (rhPTH, a genetically engineered peptide that increases bone turnover by stimulating osteoblasts) were reported for bone-TE [40]. The obtained spherical particles (~1 µm) with wrinkle-like surfaces allowed an rhPTH entrapment efficiency of 60.36–74.9% and a gradual release profile reaching around 70% after 96 h. A significant finding was that 50% of rhPTH was released at pH 7.5 within 24 h, indicating a promising sustained release capability suitable for therapeutic applications in treating bone diseases, including osteoporosis and bone fractures. The hemolysis assay showed that there was negligible damage to the blood cells associated with the obtained microparticles [40].

The desolvation method (which involves gradually removing the solvent from the emulsion) is also a straightforward approach to obtaining SF microparticles. Using this technique, Pham and co-authors [103], obtained SF microparticles loaded with *Wedelia trilobata* L. flower extract (WT), used as a strong antioxidant in Vietnamese folk medicine. The drug release profile of WT was dependent on the SF concentration and extraction solvents. The profile exhibits a two-phase release pattern: a burst release phase, where the polyphenols encapsulated in the particles are released into the medium, and a gradual release phase, which is related to the hydrophobic interactions of polyphenols with SF-II β-sheet structure. The maximum polyphenol release varied based on SF concentrations and extraction solvents, showing percentages of 26.94%, 23.67%, and 17.38% for methanol extracts; 26.77%, 23.18%, and 19.87% for ethanol 60% extracts; and 32.98%, 28.3%, and 20.62% for ethanol 96% extracts at fibroin concentrations of 1%, 2%, and 3%, respectively.

The burst release mechanism enables the delivery of an appropriate amount of drug for therapeutic purposes while also gradually extending the therapeutic effects and providing polyphenol protection. The microparticles possessed high-actions comparable to standard ascorbic acid [103].

Another approach reported the fabrication of composite microparticles composed of SF and hollow mesoporous silica nanocarriers (HMSN) generated by drying microfluidic emulsion templates of HMSN-dispersed SF solution [104]. The spherical structure with a rough surface (400 µm) was composed of two barriers that control the drug release: (1) the encapsulated HMSNs increase the drug-carrying capacity of the microparticles of dimethyloxaloylglycine (DMOG, a small molecular drug stimulating neovascularization) and form the first barrier via physical absorption, and (2) the SF microparticles provide a shell with monodispersity and biocompatibility and form the second barrier via efficient encapsulation. Loaded microparticles released approximately 70% of the loaded DMOG within 3 days, with a notable initial burst release of about 40% in the first 8 h. The particles exhibited a sustainable and prolonged release pattern over 30 days without an initial burst. The in vitro study demonstrated that the particles could induce the tube formation of human umbilical vein endothelial cells (HUVECs) while maintaining the viability and proliferation of 3T3 cells. Moreover, when sprayed onto a porcine acellular dermal matrix and implanted into a rat model with an abdominal wall defect comprised of the excision of the external and internal oblique muscles, the particles promoted new blood vessel formation via controlled release of DMOG.

Other complex and innovative strategies have been implemented to obtain SF-microparticles, including advanced micro-patches and coating systems. Microneedle patches loaded with SF microparticles (13.2 μm) for sustained transdermal delivery of levonorgestrel (contraceptive) were proposed by Yavuz et al. [105]. To achieve this, after producing the particles (through a multi-step process beginning with the isolation of silk fibroin from Bombyx mori cocoons, followed by dissolution in a LiBr solution, dialysis, and centrifugation to remove aggregates. The resulting silk fibroin solution was then used to prepare microparticles (MPs) by mixing with poly(vinyl alcohol) (PVA), casting into films, and undergoing drying, peeling, dissolution of PVA, and lyophilization), incubation with levonorgestrel was carried out before drop casting with SF solution (Figure 3C). Sustained drug release reached up to 100 days, with a cumulative release of 51.4 ± 1.1% at the end of 3 months when the drug was loaded directly inside the microneedles, while release continued for more than a year when the drug was loaded inside microparticles before casting inside the microneedle patches [105]. The addition of a surfactant and a solubility enhancer, specifically Tween-20 (1%) and β-cyclodextrin (B-CD, 5%), was found to modify the release profiles further. Both additives led to similar release profiles, with a cumulative release of 61.2 ± 1.1% and 62.1 ± 1.7%, respectively, providing enhanced release compared to formulations without these additives.

For articular surface cartilage regeneration, Zhang and colleagues [106] developed a paint for topical treatment composed of N-(2-aminoethyl)-4-(4-(hydroxymethyl)-2-methoxy-5-nitrosophenoxy) butanamide-coated SF microparticles (100–150 μm). Butanamide-modified SF microparticles can directly adhere to the cartilage and form a smooth layer on the surface via the photogenerated aldehyde group of Butanamide reacting with the −NH_2_ groups of the cartilage tissue. Paint treatment showed a significant promotion of cartilage regeneration and restored the smooth joint surface at 6 weeks post-surgery in a rabbit model of a partial-thickness cartilage defect (Figure 3D).

SF microparticles are also used to obtain three-dimensional scaffolds for applications that require load-bearing capacity (such as bone regeneration and bone void filling). The particles can be fused in a cylindrical mold using an aqueous solution to form the scaffolds [107,108] or by using a microparticle aggregation method [109].

### 2.2. Silk-Based Nanoparticles

The success of topical treatments for skin-related disorders is highly dependent on the amount of time that the topical treatment remains on the surface of the site of inflammation, which commonly leads to complications if the site of inflammation is in a challenging location or if the treatment is in a more liquid form. Additionally, poor penetration of the drug through the stiff layers of skin is often responsible for poor therapeutic efficiency [110].

Biopolymer-based nanoparticles have shown great promise in the field of dermatology for treating skin diseases and promoting wound healing. SS- or SF-based nanoparticles can be designed to encapsulate drugs or therapeutic agents, allowing for targeted delivery to specific skin layers or cells. These nanoparticles provide a controlled release of therapeutic agents, ensuring a sustained and prolonged effect, which is crucial for treating chronic skin conditions.

The small size of nanoparticles facilitates penetration through the stratum corneum, the outermost layer of the skin, improving the absorption and bioavailability of therapeutic agents [111]. The recent focus on transdermal drug administration using particle formulations for disease treatment explores the potential for systemic or local delivery of therapeutic agents through the skin, bypassing the liver’s first-pass effect [111].

Similar to microparticles, various methods have been employed to generate silk-based nanoparticles, including silk-PVA blends [112], spray drying [113], and nanoprecipitation [114,115], among others [114]. Nanoprecipitation, also known as desolvation, has become particularly popular for its simplicity in producing nanoparticles from protein solutions. This technique involves two miscible solutions, with the first solvent containing the polymer (the solvent) and the second solvent lacking it (the non-solvent). The process entails the rapid dissolution of the polymer, leading to the precipitation of nanoparticles upon the addition of the polymer solution to the non-solvent [116]. This phenomenon is driven by the Marangoni effect, where interfacial turbulences between the solvent and non-solvent govern particle formation [117].

#### 2.2.1. Physiochemical Properties Govern Nanoparticle Uptake

A nanoparticle’s behavior is governed by both its structural and chemical properties, with particle size being among the most important properties that determine targeting ability, cell uptake, and drug release. Although dependent on the material for skin-related drug delivery, smaller nanoparticles have, in general, exhibited higher cell uptake and deeper penetration compared to larger microparticles, leading to faster drug release and wider drug distribution [118,119].

Nanoparticles cannot simply diffuse across the cell membrane; they are internalized through endocytic mechanisms (pinocytosis, endocytosis), which largely depend on the physiochemical properties of the nanoparticle [120]. After internalization, nanoparticles are transported from the early endosome to the late endosome and then to the lysosomes to be broken down. In the event that nanoparticles can escape either the endosomes or lysosomes, they are released into the cytoplasm. For the delivery of mRNA, nanoparticles must escape the endosome or lysosome and enter the cytoplasm to be effective [121]; in the case of a goal to induce cell death, such as for cancer treatments, it is favorable for nanoparticles to accumulate in the lysosomes, causing rupture of the acidic lysosome and, therefore, apoptosis, or release of chemotherapy from the nanoparticle into the cell via pH responsiveness [115,122].

Previously, 100 nm SF nanoparticles (SF-NPs) were successfully generated using low-molecular-weight SF loaded with DOX for treating a human breast cancer cell line [115]. These nanoparticles were confirmed to colocalize in lysosomes, highlighting their potential for cancer treatment [123]. Using isopropanol as the non-solvent, researchers investigated the effect of stirring rate on nanoparticle production and produced particles ranging in size from 104 to 134 nm [114]. More recent work built on this concept, demonstrating that nanoprecipitated SF-NPs can be formed consistently across a wide size range from 45 to 250 nm while maintaining a low polydispersity index (PDI) of around 0.2–0.4 [124].

Silk characteristics (e.g., molecular weight and concentration) and production factors (e.g., temperature and stir speed) can be used to alter and control the size of SF- and SS-nanoparticles [124,125]. Furthermore, the surface properties of these nanoparticles (charge modification, antibody decoration) can be modified by using pre-functionalized silk as the initial precursor molecules or by introducing suitable pendant groups to the surface via a post-functionalization process. SF nanoparticles of diverse sizes were effectively tested with a cancer cell line (glioblastoma) to assess cellular uptake, serving as a model for investigating these particles in oncological applications. The entrapment of DOX in the SF nanoparticles was also explored and compared with coatings (adsorption) of the DOX on the surface of the SF nanoparticles [124]. SS-based particles have been investigated for lysosomal delivery of cargo such as DOX [126], and SS-NPs have been manipulated to display different surface charges through various methods, such as the addition of poly-L-lysine coatings [127].

Physicochemical property control is crucial for nanoparticles, influencing their interactions with biological systems, cellular uptake, drug release kinetics, and immunogenicity. With protein-based systems like SF- and SS-nanoparticles, these aforementioned studies demonstrate that precise control over the physico-chemical properties of the particles has been well established, showing their potential advantages for their use in skin-related applications.

#### 2.2.2. Silk Fibroin Nanoparticles for Delivery to the Skin

While polymeric nanoparticles have demonstrated significant promise in skin delivery, they tend to accumulate on the skin surface, in furrows, and in hair follicles without penetrating the stratum corneum [128]. Consequently, there has been an exploration of strategies that involve combining polymer nanoparticles with active enhancement techniques to improve transdermal drug permeation [129]. Active methods to enhance drug permeation encompass iontophoresis, electroporation, ultrasound, and microneedle treatment [129]. While these approaches can, to some extent, facilitate percutaneous drug penetration, certain issues persist. For instance, microneedles may induce skin inflammation when left in the skin, and electroporation can cause damage by disrupting skin structure. Magnetic and electromagnetic fields also emerge as effective approaches for augmenting drug permeation across the stratum corneum [129].

Recent advancements emphasize the significant role of hair follicles in transdermal permeation, indicating that the application of massage positively influences follicular penetration [130]. Mechanical massages have been found to allow for considerably deeper nanoparticle penetration into the hair follicles compared to cases without massage [130]. As a result, a synergistic strategy for transdermal drug delivery was presented by combining magnetic nanoparticles with an alternating magnetic field. This hypothesis holds that the alternating magnetic field affects both the organized structure of the stratum corneum (SC) lipid bilayers and the movement of magnetic nanoparticles within the epidermis, mimicking the effects of massage [130].

**Figure 4:**
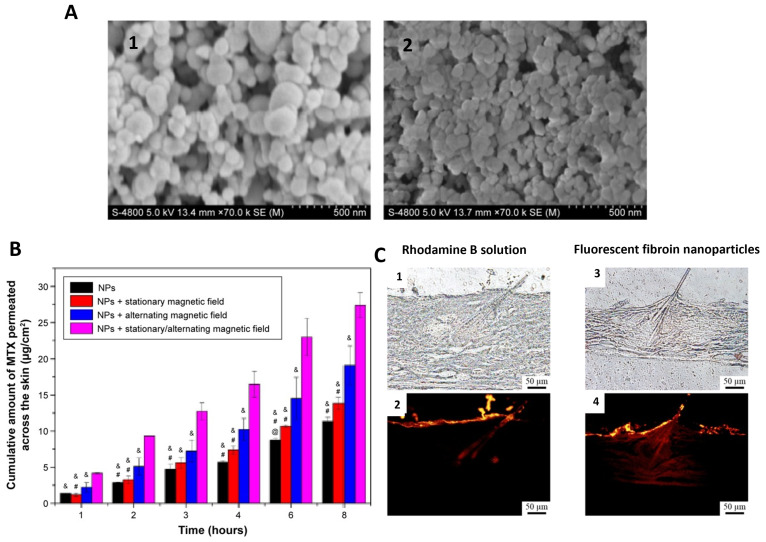
SF-based nanoparticles systems: (**A**) Non-MTX-loaded particles (**1**) and MTX-loaded Fe_3_O_4_-SF nanoparticles (**2**) (adapted from [129], Copyright 2015 Chen et al. This work is published by Dove Medical Press Limited); (**B**) MTX-loaded magnetic SF-NPs, cumulative permeation of MTX across the skin in the presence of pulsed, alternating magnetic field. @, #, and & indicate the statistical significant difference when compared with the stationary magnetic field, the alternating magnetic field, and the stationary/alternating magnetic field, respectively. *p* < 0.05. (reprinted from [129], Copyright 2015 Chen et al. This work is published by Dove Medical Press Limited); (**C**) fluorescence microscope image of a cross-section of the mouse skin after 6 h comparing the control rhodamine B solution (**1**,**2**) and the fluorescent SF-nanoparticles (**3**,**4**) (Adapted from [111], copyright 2018 Elsevier B.V.).

Methotrexate (MTX), a drug used in treating psoriasis and rheumatoid arthritis at lower doses and leukemia at higher doses, has been associated with various side effects, such as hepatic toxicity, thrombocytopenia, anemia, and fatigue when taken systemically [129]. Given these side effects, topical, local delivery of MTX is preferred; however, inadequate percutaneous absorption has been reported [129]. To overcome this, various strategies, including iontophoresis and penetration enhancers, have been explored, albeit with drawbacks such as irreversible skin damage and irritation caused by certain penetration enhancers like surfactants [129]. Chen et al. examined the use of MTX-loaded magnetic SF nanoparticles under an alternating magnetic field in excised guinea pig skin mounted in a Franz diffusion cell. Particles were fabricated using suspension-enhanced dispersion by the supercritical CO_2_ technique, combining Fe_3_O_4_ nanoparticle solution with SF-HFIP solution. Non-MTX-loaded particles had a particle diameter of 112 nm, while MTX-loaded particles had a particle diameter of 75 nm (Figure 4A). The permeability of the MTX solution through the skin was compared to the MTX-loaded magnetic SF-NPs, and it was found that the SF-NPs enhanced the drug permeation across the skin, especially in the presence of a pulsed, alternating (massage-like) magnetic field, indicating their capability for delivering drugs to the deeper layers of the skin (Figure 4B) [129].

In a previous study by Makino et al., poly(DL-lactide-co-glycolide) (PLGA) nanoparticles with an average size of 50 nm were prepared, showing limited penetration beyond the stratum corneum [131]. This led to the investigation of SF as a base material for nanoparticles due to its potential higher delivery properties to deeper skin layers [111]. In this study, SF-NPs with an average size of 45 nm or less were prepared using an antisolvent diffusion method; SF fibers were dissolved in a LiBr solution with heat, cooled to room temperature, diluted with water, and then centrifuged to remove insoluble debris [111]. The obtained SF solution was then injected into an acetone solution and then dialyzed into a NaCl solution to remove the acetone [111]. Physicochemical characteristics and storage stability were evaluated, where rhodamine B-labeled particles demonstrated a ~36 nm particle size and non-fluorescently tagged particles were ~42 nm with low PDIs. Both versions of the prepared nanoparticles showed similar stable structure and size over time at various temperatures [111]. Mouse skin penetration studies revealed that when comparing the control rhodamine B solution and the fluorescent SF-nanoparticles, the rhodamine B solution primarily reached the stratum corneum and hair follicles, with limited penetration into surrounding tissues (Figure 4C). In contrast, fluorescent SF-nanoparticles penetrated the stratum corneum, hair follicles, epidermis, and dermis within 6 h of administration, suggesting their potential for drug delivery to deeper skin layers for skin-related diseases or wound healing applications [111].

In another study, the optimal size profile of SF-nanoparticles was determined for transdermal delivery. Silk nanofiber size was gradually reduced from 2000 to <100 nm and investigated for their capacity to permeate through the skin. Particles were fabricated first using a concentration-dilution protocol [132], where SF solution was concentrated while being heated over the course of 24 h to form particles, then diluted and heated until a gel was formed. An ultrasonic treatment was then employed between 100–650 W for 5–30 min to prepare silk nanoparticles of different sizes by altering the ultrasonic intensity and time. A Franz diffusion cell using ex vivo human skin was utilized to test particle permeation through the skin in vitro, while a rat model was utilized for in vivo studies [132]. Particle length decreased with the increased intensity of the ultrasonic treatment. Curcumin-loaded silk nanofibers were applied to the dorsal skin of rats for 18 h, with and without carbomer, to facilitate particle spreading on the skin. Controls administering free curcumin demonstrated that unbound curcumin itself was not able to significantly penetrate the skin. In contrast, curcumin-loaded silk nanofibers were able to deliver significantly higher amounts of curcumin to the corneum layer after 18 h, with smaller particles displaying increased and faster transdermal delivery capacity [132]. Rhodamine-B-loaded and DOX-loaded silk nanofibers were tested in the in vitro Franz diffusion cell model in human skin. Similar to the in vivo test, when compared to free drug formulations, particle formulations performed better in delivering cargo through the human skin tissue, and stronger fluorescence signals were seen in the smallest particle size (40 nm), supporting the use of SF-NPs in skin drug delivery or potential wound healing applications [132].

Using a desolvation technique, *Avicenna marina* extract and neomycin-loaded SF-nanoparticles were fabricated by Karaly et al. for wound healing applications using an aerosolized nano powder formulation as the delivery mechanism [133]. *Avicenna marina* extract was utilized for not only its antioxidant and antibacterial activity but also its ability to act as a cell proliferation enhancer for the tissue regrowth phase of wound healing. These particles were between 280 to 307 nm in diameter; however, when put into the aerosol formulation, the size increased to 715 nm. Particles were tested against *Staphylococcus aureus*, *Methicillin-resistant S. aureus*, *Pseudomonas aeruginosa*, and *resistant P. aeruginosa*, and particles displayed good antibacterial activity [133]. In scratch wound healing assays on human skin fibroblast monolayers, 100% wound closure was demonstrated after 24 h incubations of the aerosolized nanopowder formulation vs. 70% wound closure against the positive control [133]. These results were confirmed in a rodent model, where histopathological results demonstrated that after inflicting a wound area on the dorsal back of a rat, there was an increase in fibroblast proliferation and a decrease in inflammatory cells after 15 days, with total wound closure in comparison to controls. These results showed the potential benefits of this particular spray system for wound healing applications [133].

#### 2.2.3. Silk Sericin Nanoparticles for Delivery to the Skin

As mentioned, SS has been established to have antioxidant, moisturizing, and wound-healing properties. Silver-based materials have also been substantially established as materials used in wound healing applications due to their antibacterial activity; however, the staining of the skin from silver-based topical antibiotics, even years after their use [134], burning, and irritation after repeated application, present disadvantages that may limit their use [135]. Silver nanoparticles have been shown to have better antimicrobial properties than bulk metal and can potentially overcome some of the limitations seen with current solutions, but they are unstable in aqueous environments [135]. Similarly to SS, chitosan has also demonstrated wound-healing effects [135,136]. For these reasons, Verma et al. [135] investigated a sericin/chitosan-capped silver nanoparticle system for wound healing applications, where NPs were prepared via the chemical reduction of silver nitrate using chitosan and sericin as the stabilizing polymers. Chitosan solution (in acetic acid) was mixed with sericin solution (in water) until homogenous and then mixed with silver nitrate solution at varying concentrations for 24 h. The formation of the particles was confirmed when the mixture turned a dark yellow color [135]. Particles ranged from ~240 nm to ~970 nm, with larger particles being formed when higher concentrations of silver nitrate were used. Particle formulations (silver particles on their own, SS/chitosan silver particles, and SS/chitosan silver particles embedded in a Carbopol gel) were tested against *Escherichia coli* and *S. aureus*. Significant differences in the antimicrobial activity between silver particles on their own and SS/chitosan silver particles embedded in the Carbopol gel were observed for both bacterial tests. SS/chitosan silver particles without the Carbopol gel demonstrated increased antimicrobial activity in comparison to the silver particles on their own, but the inclusion of the particles inside the gel led to the highest antimicrobial activity [135]. The particles were also investigated in a rat wound model, where a wound was made on the dorsal side of the rat using a scalpel, removing the epidermal and dermal layers of the skin. Besides the untreated control group, rats were treated with either povidone–iodine treatment (positive control) or SS/chitosan silver particles embedded in a gel over 14 days. On the 7th day, particle-hydrogel-treated wounds demonstrated faster scab formation and decreased wound area than the control groups. By the 14th day, hydrogel particle-treated wounds had no signs of infection or inflammation [135]. Histological data showed that, in comparison to the other groups, the hydrogel particle-treated wounds showed increased keratin and blood vessel formation. Pandiarajan et al. also developed SS-silver nanoparticles that showed potential antibacterial and antifungal activity against virulent strains [137].

**Figure 5:**
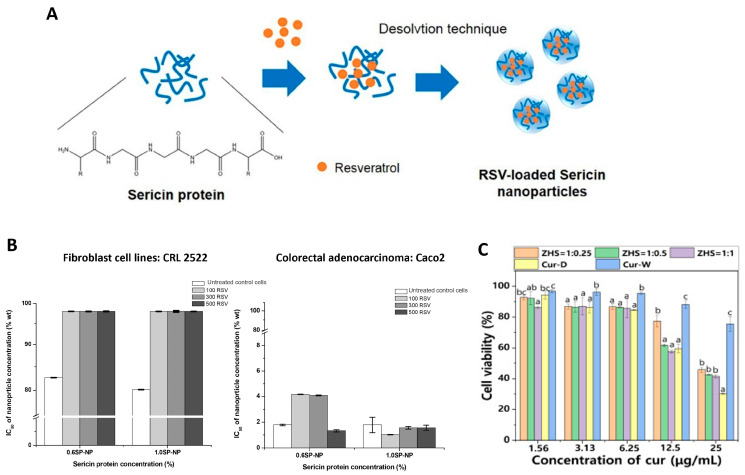
SS-based nanoparticles systems: (**A**) resveratrol-loaded SS nanoparticles via a precipitation technique (Adapted from [138], Copyright 2017 Elsevier B.V.); (**B**) the inhibition concentration at 50% (IC50) of resveratrol-loaded SS nanoparticles with normal cells and colon cancer cells (adapted from [138], Copyright 2017 Elsevier B.V.); (**C**) zein-SS nanoparticles loaded with curcumin anti dermatitis effect—cell viability of HaCaT cells exposed to nanoparticles and curcumin suspension. ZHS = zein-to-silk sericin mass ratios, Cur-D = free curcumin dissolved in DMSO, Cur-W = free curcumin dissolved in water. a, b and c indicate significant differences (*p* < 0.05). (Adapted from [139], Copyright 2022 Elsevier Ltd.).

In another study, resveratrol-loaded SS nanoparticles were fabricated using a nanoprecipitation technique (Figure 5A). and tested in human skin fibroblasts and colorectal adenocarcinoma cells [138]. SS powder was added to water at varying concentrations. The SS solution was dropped through an atomizer into a solution of Pluronic F-68 in dimethyl sulfoxide under stirring. All formulations resulted in particle sizes between 200–350 nm, with increased sericin concentration producing larger particles and increased surfactant concentration resulting in smaller particles [138]. When particles were loaded with or without resveratrol and incubated with human skin fibroblasts and colorectal adenocarcinoma cells for 24 h, more than 80% of the skin fibroblasts maintained their viability, while all particle conditions demonstrated cytotoxicity in the colorectal adenocarcinoma cells (Figure 5B).

A more recent study on zein-SS nanoparticles loaded with curcumin was carried out for the treatment of atopic dermatitis, which occurs when leukocytes migrate into the dermis due to increased chemokine production at an inflammation site [139]. Typical topical treatments such as gels, creams, and ointments are inadequate due to insufficient penetration of drug molecules into the skin [139]. To fabricate the particles, zein was dissolved in ethanol solution, while sericin was added to water. Zein solution was injected into the sericin solution at pH 9 under stirring for 2 min, and the ethanol was removed using rotary evaporation. Particles were between 330–400 nm in diameter, with more SS used in the formulation contributing to smaller particle sizes [139]. Quantitative skin penetration assays were performed using a Franz diffusion cell system with a Strat-M membrane as an artificial human skin equivalent and ex vivo porcine skin (with removed hair) for 24 h incubation with the particles. Cytotoxicity and cellular uptake tests were also performed with HaCaT cells, which are a human keratinocyte line [139]. These tests indicated that these particles were able to deliver curcumin across the epidermis and reach deeper sites within the skin better than the free curcumin control. These particles also downregulated the production of inflammatory cytokines in keratinocytes, demonstrating an anti-dermatitis effect (Figure 5C) [139].

#### 2.2.4. Protein Corona Is Still Unexplored in Skin-Related Particle Systems

As mentioned, naturally derived biopolymer-based NPs, specifically protein-based NPs, have many intrinsic characteristics that make them advantageous for targeting certain cell types. For example, they can be both genetically and chemically modified to display cell-targeting ligands on their surface [140]. Depending on the protein, they can be tuned to express a positive or negative surface charge, as cells are slightly negatively charged, and some therapeutics can be ionically bound to the surface of NPs based on charge alone [140]. In vivo, these surface charges will change largely based on the protein corona, which describes the layer of proteins that assemble on the surface of the particle after the introduction of the particle to physiological fluids [141]. This protein layer is governed by the physicochemical properties of the nanoparticle, such as surface charge and hydrophobicity, and it greatly impacts how readily the particles are cleared by the immune system; adsorbed immunoglobulins on the particle surface can result in them being cleared by the immune system, for example [141]. Therefore, the protein corona is critical for understanding and controlling cellular uptake and reaching target tissues.

With protein-based particles, namely SF or SS, surface charge and degree of hydrophobicity can be more readily tuned, allowing for potential control of the protein corona in vivo [140,142,143]. Without characterizing the protein corona, the translation of targeted drug delivery using NPs from bench-to-bedside becomes challenging [144]. The significance of the protein corona varies depending on the target organ or area of the body. Specifically, when considering the skin, the protein corona plays a pivotal role in nanoparticle interactions and responses.

Skin, being the outermost barrier of the body, interacts significantly with external agents. External factors, such as exposure to sunlight, sweat, or environmental pollutants, may alter the composition of the protein corona in nanoparticles applied to the skin. Different skin cell types or skin states (healthy vs. wounded) may respond differently to nanoparticles based on the protein corona, affecting aspects such as inflammation and allergic reactions. Understanding the protein corona’s role in cellular interactions helps in designing nanoparticles for effective skin delivery of drugs or therapeutic agents. By understanding how the protein corona affects the behavior of nanoparticles in the skin, researchers can tailor the surface properties of nanoparticles to enhance their performance for specific applications, such as drug delivery or imaging. As more silk-based particle systems are being investigated for skin-related drug delivery applications, it is important to note that investigating the protein corona in skin applications is essential for assessing potential adverse effects and ensuring the safe use of nanoparticles in dermatological treatments.

### 2.3. Silk-Based Liposomes

Silk-based liposomes are a type of nano- to micro-drug delivery system that can deliver bioactive molecules to the skin for wound healing and skin regeneration [145]. They have the ability to enclose hydrophilic drugs within their core and accommodate hydrophobic or lipophilic drugs in their lipid bilayer, which resembles cell membranes. They can offer numerous benefits, such as biocompatibility, self-assembly capabilities, and the ability to encapsulate a wide range of drugs or macromolecules. Additionally, they can be designed to control drug release in response to environmental stimuli (e.g., pH, temperature, glucose concentration) [145,146,147] and can be modified with polymers or ligands to alter their physicochemical and biophysical properties [146].

The study and development of silk-based liposomes as drug carriers have not been as explored in comparison to the other particulate systems mentioned.

**Figure 6:**
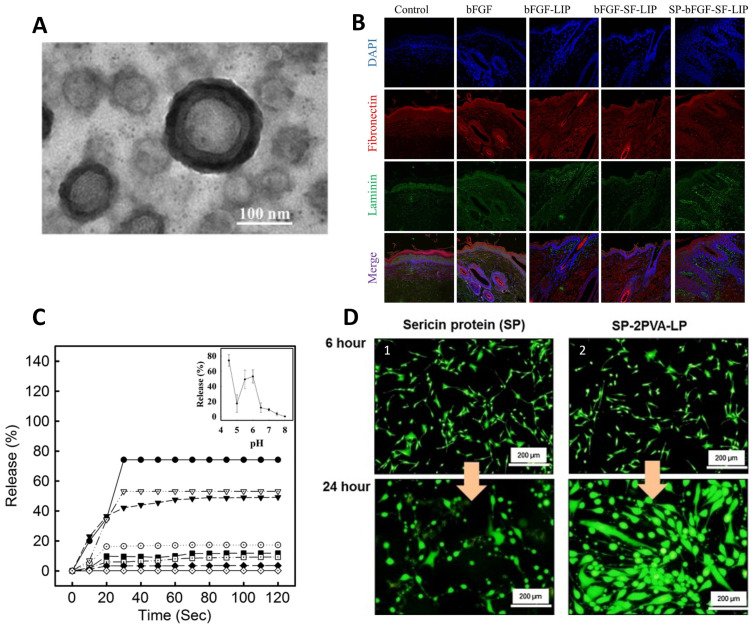
SF- and SS-based Liposome systems: (**A**) TEM image a liposome with a hydrogel core made of SF that can encapsulate bFGF (adapted from [149], Copyrights 2018 Elsevier B.V.); (**B**) immunofluorescent staining of fibronectin and laminin in wound skin at 21 days after various treatments of SF liposome encapsulate with bFGF (reprinted from [149], Copyrights 2018 Elsevier B.V.); (**C**) calcein releases profiles of liposomes mixed with HmCh and HmSF at pH 4.5 (●), pH 5.0 (○), pH 5.5 (▼), pH 6.0 (▽), pH 6.5 (■), pH 7.0 (□), pH 7.5 (◆), and pH 8.0 (◇) (reprinted from [146], Rights managed by Taylor and Francis); (**D**) confocal microscopy images of fibroblasts (CRL-2522) after incubation with SS (**1**), and SS copolymer-liposome (**2**) nanoparticles prepared with PVA (SP-2PVA-LP) for 6 h and 24 h, respectively (bar = 200 μm) (reprinted from [148], Copyrights 2016 Elsevier B.V.).

Xu et al. [148,149] demonstrated a new liposome with a hydrogel core made of SF that can encapsulate basic fibroblast growth factor (bFGF) (bFGF-SF-LIP) for wound healing (Figure 6A). To improve the permeability of the liposome without compromising encapsulation efficiency, laurocapram was added to the liposomal membrane to create a skin-permeable liposome (SP-bFGF-SF-LIP) [149]. bFGF is a growth factor that is highly beneficial in promoting hair follicle neogenesis and overseeing ECM remodeling in wound healing. However, bFGF clinical efficacy is significantly impeded due to inadequate stability in wound fluids and limited permeability through dense wound scars. The current study showed that after a 3-day incubation period with wound fluid, the formulated liposomes exhibited a retention rate of approximately 65% for bFGF, in contrast to the free drug. The studies suggested that encapsulating bFGF significantly enhanced its skin permeability, allowing it to penetrate into the dermis. This improved the morphology of hair follicles at the wound zone and promoted hair regrowth in deep second-scald mice models. Additionally, encapsulating bFGF in SF liposomes improved the growth factor’s stability when in contact with wound fluids. The deep second-degree scald wound was completely closed 21 days after treatment with the developed liposome (Figure 6B). Additionally, hair regrowth in the wound area was more pronounced in this group compared to the other groups [149]. In this line of investigation, the authors also used liposome-incorporated basic fibroblast growth factor (FGF-2) to prevent hair loss in patients with alopecia areata [150].

Several studies have examined how the pH of the environment affects the controlled release of encapsulated compounds by liposomes [147]. Hong et al. used the SF as a sensor and actuator for pH-sensitive release [147]. The authors hydrophobically modified the SF by covalently attaching palmitic acid residues. Then, liposomes containing hydrophobically modified SF (HmSF) were prepared by hydrating dry egg phosphatidylcholine film with an aqueous solution containing HmSF [147]. Also, complexation-responsive pH liposomes were developed by immobilizing HmSF and hydrophobicized chitosan (HmCh) on the surface of egg phosphatidylcholine liposomes for the release of calcein [151]. These studies suggested that liposomes maintain stability in terms of release within the pH range of 6.5–8.0 (Figure 6C). However, a release trigger was observed at pH values below 6 due to the conformational transition of HmSF. Release began at pH 5.5 and reached over 90% at pH 4.5 [147,151].

Various types of SF liposomes have been researched for different therapeutic purposes. In some cases, SF was used to coat the liposomes, which extended their circulation time and enhanced the efficacy of DOX, an anticancer drug, for longer cancer treatment [146]. Another study evaluated the drug release kinetics and therapeutic availability of silk-fibroin-coated, emodin-loaded liposomes [145] and curcumin [152]. The SF coating altered the drug release kinetics, resulting in a longer release time compared to the uncoated liposomes.

From the information presented, it can be inferred that SF is a more suitable candidate for liposomes than sericin. This is because SS is a hydrophilic protein that dissolves easily in liquid mediums, making it more difficult to control drug release. It is worth noting that SS has mainly been studied for its bioactive properties rather than as a substance suitable for controlled drug release [153]. Suktham et al. [153] developed a method for recovering SS from silk wastewater by encapsulating it with copolymer-liposome nanoparticles fabricated using the thin-film hydration method from PVA. The cytotoxicity and viability assays showed that liposomes modified with SS on the surface facilitated the attachment and proliferation of human skin fibroblasts (CRL-2522) without any adverse effects (Figure 6D). These findings demonstrate the benefits of SS encapsulation, including the ability to reduce cell death and allow for higher drug dosages during extended contact periods. The addition of PVA as a copolymer significantly improved liposome stability and increased encapsulation efficiency. Over a four-month period at varying temperatures, the protein content of sericin powder decreased to less than 10%, whereas sericin-loaded liposome powders maintained a sericin content below 20%. Furthermore, the DPPH scavenging assay demonstrated that the liposomes containing a higher amount of PVA maintained their antioxidant activity consistently, even after three months of storage [153].

### 2.4. Silk-Based Aerogel Microparticle Systems

Aerogel-based biomaterials (organic, inorganic, and composite) have unique properties such as biodegradability, high porosity (>95%), large surface area (>200 m^2^/g), high liquid absorption capacity, and outstanding textural properties, making them promising biomaterials for biomedical applications, including wound healing, bone regeneration, and drug delivery [154,155,156,157]. Aerogels are synthesized through sol–gel technology, assisted by supercritical carbon dioxide (scCO_2_, a green and sustainable technology) drying, removing liquid solvents in the gels without significantly damaging their three-dimensional-backbone, resulting in aerogel scaffolds with tunable surface properties, high loading yields and reproducibility, and controlled drug delivery [155,156,157].

Aerogel-based biomaterials have been studied as a suitable option for wound dressings, specifically due to their excellent physico-chemical and structural features, which can reduce exudation and encourage clot formation, as well as moisture and pH control at the damaged site [2]. Besides their excellent biocompatibility, biodegradability, low cytotoxicity, and good biological performance, aerogels can act as carriers for bioactive compounds (e.g., growth factors and others, proteins, cells, and antimicrobial agents), contributing to enhancing the wound healing process and preventing the colonization of microorganisms [158].

**Figure 7:**
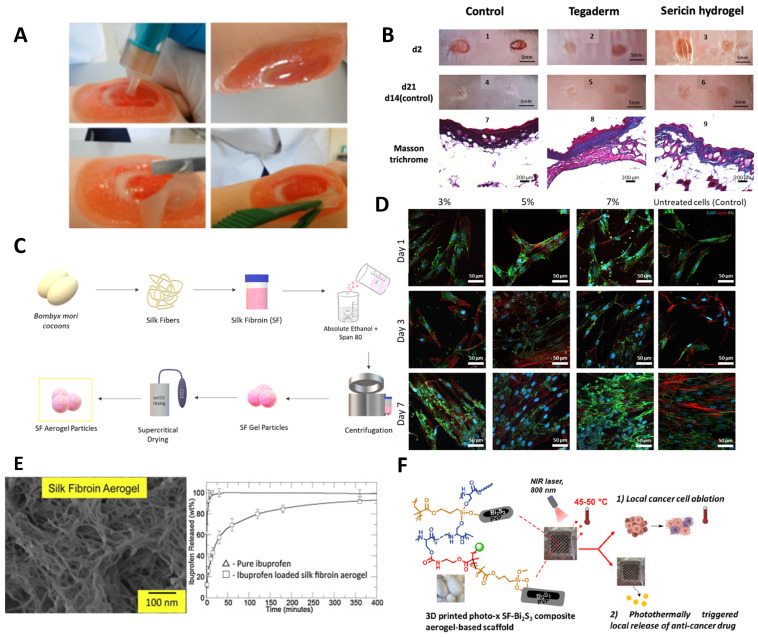
SS- and SF-based aerogel systems: (**A**) SSH applied in situ on a wound simulator and macroscopic analysis (adapted from [159], Copyrights 2022 MDPI, Basel, Switzerland); (**B**) representative images of wound closure (**1**–**6**) and histopathological analysis by Masson’s Trichrome staining (**7**–**9**) in a mouse model of a skin wound healing assay. The wounds were treated with sericin hydrogel and covered with Tegaderm, only covered with Tegaderm, or left untreated (control) (reprinted from [159], Copyrights 2022 MDPI, Basel, Switzerland); (**C**) schematic illustration of the procedure used for preparing SF aerogel particles (reprinted from [17], Copyrights 2023 MDPI, Basel, Switzerland); (**D**) confocal microscopy images were used to visualize the morphology, distribution, and growth of the nuclei, cytoskeleton, and fibronectin of human dermal fibroblasts (HDFs) when cultured in direct contact with the produced sample SF-Ap for 1, 3, and 7 days of culture (reprinted from ([17] Copyrights 2023 MDPI, Basel, Switzerland); (**E**) SEM images of SF-aerogel and in vitro ibuprofen release in PBS at 37 °C and pH 7.4 over a 6-h (reprinted from [163], Copyright 2014 Elsevier B.V.); (**F**) schematic image of three-dimensional aerogel-based composite scaffold combining the therapeutic and regeneration functionalities for successive tumor therapy and tissue regeneration (reprinted from [164], Copyright 2023 American Chemical Society).

As previously stated, the silk of silkworms has multiple potential applications in biomedicine due to its good biocompatibility, mechanical properties, degradability, and plasticity. Several studies have shown that silk-based material dressings are more successful than traditional ones in the healing of skin wounds [34,159,160,161]. For instance, Batista-Silva et al. [159] developed a novel in situ-forming SS hydrogel as an enhanced dressing for wound healing using a simple technology based on horseradish peroxidase crosslinking (Figure 7A). The authors observed that the SS-hydrogels promoted wound closure and a decrease in granulation tissue compared to commercial Tegaderm^TM^ dressing (Figure 7B). The hydrogel also enhanced the deposition of collagen fibers with smaller diameters, positively affecting re-epithelialization. Moreover, no inflammatory reaction was observed (Figure 7B). In addition, the authors assessed the SS hydrogel’s antioxidant potential during in vitro degradation under physiological protease concentrations. The antioxidant activity increased during the breakdown process, demonstrating that crosslinking with horseradish peroxidase and hydrogen peroxide had no effect on this activity. The highest levels of antioxidants were obtained at 24 h, which could be attributed to the release of phenolic compounds. It should be noted that antioxidant activity can be an important factor in limiting the oxidation of inflammatory processes during healing and tissue regeneration events [159].

Mallepally et al. showed that SF aerogel scaffolds produced by the scCO_2_-assisted acidification method had a higher pore volume and a five-fold higher surface area compared to SF cryogels. These findings revealed that an SF-aerogel scaffold allowed for the human skin fibroblast cells’ attachment and viability, as well as a uniform distribution of cells within the aerogel. That allowed us to conclude that this scCO_2_ method can be used to produce three-dimensional aerogel scaffolds with unique features and may also be utilized for many TE applications, such as cell differentiation in controlled microenvironments [162].

SF aerogel particles were produced through the addition of ethanol during gel formation followed by scCO_2_-assisted drying, with enhanced biological properties (Figure 7C) [17]. Different SF concentrations were used to investigate the potential of this technology to produce size- and porosity-controlled particles. The results revealed that high SF concentrations enhanced the viscosity of the SF precursor solution, allowing for the formation of larger particles with a broader size dispersion. In addition, the aerogel particles had a mesoporous interconnected structure, which aided cell movement and nutrient transfer. SF aerogels showed biocompatibility, promoting cell viability, attachment, and proliferation, showing their potential for various biomedical applications—particularly in tissue healing and regeneration (Figure 7D). Moreover, SF aerogel particles displayed significant antioxidant activity and sustained degradation in the presence of protease enzymes, making them promising systems for wound healing and tissue regeneration applications [17].

The potential of SF aerogels as drug-delivery devices has been investigated [163,164]. For instance, Marin et al. produced ibuprofen-loaded SF aerogels using scCO_2_ to demonstrate the performance of these aerogels for controlled delivery applications. The in vitro ibuprofen release from SF aerogels occurred over a 6 h period and was modeled using the Ritger–Peppas model, indicating that ibuprofen release followed Fickian diffusion. It should be highlighted that the concentration and molecular weight of SF in solution had a considerable influence on SF sol–gel kinetics as well as the textural and morphological features of aerogels (Figure 7E). Despite this, the results showed the ability of SF aerogels to deliver a model drug for an extended time [163]. Another recent work developed a multifunctional all-in-one biomaterial combining the therapeutic and regeneration functionalities for successive tumor therapy and tissue regeneration (Figure 7F). In this study, an SF aerogel scaffold with a dual-network structure was successfully fabricated, combining the properties of photothermally triggered controlled anticancer drug release and photothermal cancer cell ablation. The developed scaffolds presented hierarchically organized porosity and excellent photothermal conversion thanks to the strong near-infrared photon absorption of incorporated nanobelts inside the scaffold matrix. The drug release profile of sorafenib from aerogel-based scaffolds was determined by irradiating the scaffolds with an NIR laser at different pHs (4 and 7). The drug release values were pH-dependent; at high pH, the drug release was faster, and even after 400 min of irradiation time, a release of sorafenib still took place. This was attributed to the surface charge of sorafenib and the scaffold, which becomes positively charged when pH levels rise, facilitating drug release. Thus, the heat generated in the scaffold, mediated by laser irradiation, triggered controlled and prolonged release of the anticancer drug, which significantly affected the viability of the bone cancer cells adhered to the scaffold. In addition, the aerogels have exhibited outstanding biodegradability, allowing them to be employed as possible implants to be replaced by de novo tissue [164].

## 3. Conclusions

Silk-based microparticles, nanoparticles, and liposomes have been widely studied over the last years as they provide a powerful and versatile platform for the design of functional biomaterials; presently, the know-how on SF is significantly higher than on SS, which started to be upcycled for biomedical engineering in the last decade. This is reflected in the extension of work and publications on SF as compared with SS particles. SF mechanical properties enable their use for bone and cartilage regeneration, filling of larger bone defects, and transdermal robust delivery systems. Moreover, SF can be used as stable microcarriers [41,104], to grow adherent cells and be further used to obtain organoids and mature micro-tissues [165]. SS particulate systems have been widely utilized in drug delivery and skin wound treatment, leveraging their bioactive properties, notably antioxidant features and encapsulation ability [166].

Microparticle systems, despite their lower surface-to-volume ratio as compared to nanoparticles, tend to display a decreased burst release due to reduced immediate surface contact with the environment. This, coupled with the higher encapsulation efficiency, mitigates the likelihood of burst release. Nevertheless, the absence of in vivo and subsequent clinical trials for the systems developed so far prolongs the process of transitioning SS products to the market [49].

Silk-based liposomes have potential as drug carriers for wound healing and skin regeneration. SF and SS have been studied as stabilizers for drugs in liposome formulations. Liposomes improve skin permeability, enhance stability in wound fluids, significantly increase drug efficacy, and control the release rate of the drug [145,146,153]. Liposomes have not been extensively studied for SS-controlled drug release due to their hydrophilic nature, which favors SF instead. However, recent methods, such as SS encapsulation in copolymer-liposome nanoparticles, have shown promising results.

The inherent characteristics of silk-based nanoparticles offer unique advantages for targeting specific cell types. Manipulating silk characteristics enables the alteration and control of the size of SF- and SS-nanoparticles. Moreover, these nanoparticles made of silk have the potential to improve the skin’s absorption of drugs compared to free drugs. This suggests that they could be useful for delivering a drug to deeper layers of the skin, especially in cases related to skin disease or wound healing [111].

Aerogels are considered promising carriers for bioactive compounds in the biomedical field. The research on SF aerogels as drug delivery devices demonstrates their potential to improve the wound healing process and prevent microbial colonization [158]. To our knowledge, no studies have been conducted on the use of SS as aerogel particles for skin regeneration. The interconnected mesoporous structure of aerogel particles has been demonstrated for SF, which further facilitated cell movement and nutrient transfer [17]. This highlights the potential significance of aerogel technology in advancing therapeutic strategies for effective wound healing.

A diversity of drug candidates and advanced preparation techniques, including nanoparticle encapsulation and hydrogels, are now revolutionizing wound healing and skincare. These methods enable targeted delivery of therapeutics tailored to specific needs like infection control and inflammation reduction, resulting in improved outcomes and faster recovery. Silk-based drug release systems are particularly promising in this area. They can be designed to stabilize an enclosed drug, exhibit stimuli-responsive drug release, or present different skin permeation according to particle structure and properties.

Silk processing versatility allows for optimizing size, surface properties, and drug-loading capacities to precisely target specific therapeutic outcomes. However, bridging the gap between laboratory research and clinical translation remains essential. Conducting comprehensive in vivo studies and advancing to clinical trials is necessary to validate the efficacy and safety of the most promising silk-based particulate systems in real-scenario applications, creating the path for regulatory approval and commercialization.

## Figures and Tables

**Table 1:** Fibroin’s most relevant properties in the field of biomedical engineering.

Properties	Observations	References
Antioxidant activity	SF exhibits significant antioxidant activity due to the presence of specific amino acids and an abundance of other chemical groups (hydroxyl and carboxyl).	[17,22]
Anti-inflammatory	Suppression of the elevated levels of cyclooxygenase (COX)-2, interleukin (IL)-6, IL-1β, and tumor necrosis factor (TNF)-α; stimulation of IL-10.	[23,24,25,26]
Antibacterial	SF naturally lacks inherent antibacterial activity, requiring the combination of anti-bacterial agents.	[27,28]
Metabolic regulation activity (anti-diabetic)	SF exerts anti-diabetic effects by increasing pancreatic β cell mass in a non-insulin dependent diabetes mellitus mouse model and enhances insulin secretion.	[29,30]
Collagen production	SF promotes type-III collagen.	[31]
Mimic the organic component in bone-like systems	Scaffold-based frameworks made of SF can be employed to recreate the biomineralization process of bone tissue. SF serves as a supportive structure for the proliferation of osteoblasts.	[32,33]
Moisturizing	SF shows great promise as a natural moisturizing agent.	[26]
Promotion of cell behavior	SF promotes cell growth and migration of keratinocytes and fibroblasts, enhances pro-angiogenic activity, and considerably expedites the healing of skin wounds.	[17,34]
Anti-tumor	Gamma-irradiated SF reduces the tumor growth in B16BL6 (mouse melanoma cancer).	[35]
Biodegradability	SF is susceptible to biological degradation by proteolytic enzymes (e.g., chymotrypsin, actinase, protease XIV, carboxylase).	[36,37,38]

**Table 2:** Sericin’s most relevant properties in the field of biomedical engineering.

Properties	Observations	References
Antioxidant and Photoprotective Activity	SS exhibits ROS scavenging activity protects cells from oxidative stress and shows suppression of lipid peroxidation. The antioxidant activity of SS benefits from its high serine and threonine content.	[59,60,61]
Anti-inflammatory	Suppression of the COX-2 enzyme and nitric oxide production.	[62,63]
Antibacterial	Inhibitory effects on *Staphylococcus epidermidis*, *Staphylococcus aureus*, and *Bacillus subtilis*. The reason is that SS has a positive amino acid side-chain due to its carboxyl group being protonated under acidic conditions, thus having antibacterial activity.	[60,64,65,66]
UV protection	SS reduces UVA and UVB radiation-induced skin damage.	[67,68]
Metabolic regulation activity (anti-diabetic)	As a dietary additive, SS is demonstrated to regulate glycolipid metabolism.	[69]
Collagen production	SS from all strains promotes type-I collagen production in a concentration-dependent manner.	[70,71]
Mimic the organic component in bone-like systems	It can be used to study the biomineralization process of bone tissue or to develop bone-like structures for bone-TE.	[52,72]
Moisturizing	SS has a hygroscopic (water-attracting) nature, which allows it to attract and retain moisture.	[73,74]
Supplement	SS can be used as a cell culture medium supplement or substitute due to its nutrient content and reduced immune response.	[75,76,77,78]
Promotion of cell behavior	Promote cell growth of different mammalian cell lines.	[48,79,80]
Anti-tumor	Inhibition of tyrosinase and polyphenol oxidase activities.	[46,81]
Biodegradability	Ability to be broken down: degradation is mediated both in vitro and in vivo by proteolytic enzymes (e.g., protease XIV, α-chymotrypsin, proteinase K, papain, matrix metalloproteinases, and collagenase) which act on the amorphous hydrophilic segments.	[37,82]
